# Robust optimization of the Gross Tumor Volume compared to conventional Planning Target Volume-based planning in photon Stereotactic Body Radiation Therapy of lung tumors

**DOI:** 10.2340/1651-226X.2024.40049

**Published:** 2024-06-20

**Authors:** Thomas L. Fink, Charlotte Kristiansen, Torben S. Hansen, Torben F. Hansen, Rune S. Thing

**Affiliations:** a Department of Oncology, Lillebaelt Hospital, University Hospital of Southern Denmark, Vejle, Denmark; b Institute for Regional Health Research, University of Southern Denmark, Odense M, Denmark

**Keywords:** Stereotactic body radiation therapy, dose planning, isodose volume, organ at risk, tumor motion, plan comparison

## Abstract

**Background:**

Robust optimization has been suggested as an approach to reduce the irradiated volume in lung Stereotactic Body Radiation Therapy (SBRT). We performed a retrospective planning study to investigate the potential benefits over Planning Target Volume (PTV)-based planning.

**Material and methods:**

Thirty-nine patients had additional plans using robust optimization with 5-mm isocenter shifts of the Gross Tumor Volume (GTV) created in addition to the PTV-based plan used for treatment. The optimization included the mid-position phase and the extreme breathing phases of the 4D-CT planning scan. The plans were compared for tumor coverage, isodose volumes, and doses to Organs At Risk (OAR). Additionally, we evaluated both plans with respect to observed tumor motion using the peak tumor motion seen on the planning scan and cone-beam CTs.

**Results:**

Statistically significant reductions in irradiated isodose volumes and doses to OAR were achieved with robust optimization, while preserving tumor dose. The reductions were largest for the low-dose volumes and reductions up to 188 ccm was observed. The robust evaluation based on observed peak tumor motion showed comparable target doses between the two planning methods. Accumulated mean GTV-dose was increased by a median of 4.46 Gy and a non-significant increase of 100 Monitor Units (MU) was seen in the robust optimized plans.

**Interpretation:**

The robust plans required more time to prepare, and while it might not be a feasible planning strategy for all lung SBRT patients, we suggest it might be useful for selected patients.

## Introduction

Stereotactic body radiation therapy (SBRT) for small lung tumors is established as the standard non-surgical way of treating pulmonary malignancies of up to 5 cm with curative intent [1]. Dose and fractionation varies greatly between countries and even institutions, with a mean Biologically Effective Dose for α/β = 10 (BED10) > 100 Gy being the commonly accepted goal for a curative treatment [2]. In Denmark, the mostly used fractionations are 66/67.5 Gy or 45 Gy in three fractions prescribed to the Gross Tumor Volume (GTV) [3]. Until recently, 45 Gy were mainly used for metastases, frail patients, and/or tumors in difficult locations not possible to treat with 66/67.5 Gy. Following recent national consensus, 66/67.5 Gy is now the standard treatment for all patients, where 45 Gy is only offered when 66/67.5 Gy is not safe.

According to the International Commission on Radiation Units and Measurements (ICRU) definitions [4], the GTV defines the visible extent of the tumor. Following the ICRU 91 on SBRT treatment, a margin is added to the GTV to account for internal uncertainties, like organ motion, hereby forming the Internal Target Volume (ITV). To this volume another margin is added to account for external uncertainties, such as setup errors, hereby forming the Planning Target Volume (PTV). The required minimum margins to achieve the intended dose to the GTV have been studied extensively [5], and no consensus has been reached.

The intended treatment dose may be prescribed to the GTV, the PTV, or to both volumes. Prescription practices vary between institutions, and may be based in tradition, local experience, and other reasons. This can make it difficult to compare the results of others with one’s own practice [6], but to facilitate these comparisons, reporting of both PTV and GTV doses are recommended by the ESTRO/ACROP guidelines on SBRT [1].

Robust optimization is a type of minimax optimization algorithm capable of optimizing a radiation treatment plan to ensure delivery of the prescribed dose across a range of scenarios, such as different movements of the target tumor [7]. This method has become the standard when planning proton therapy making it possible to handle the very different dose deposition with protons due to the Bragg-peak. This dose-depth profile means that a PTV is not able to ensure the intended dose coverage, because of the very different tissue densities between the tumor and the surrounding lung tissue, in other words the static dose cloud approximation does not hold true [7]. With robust optimization deployed for proton planning, its application for photon planning seems relevant to investigate [8].

The use of robust optimization in photon therapy planning is limited. Previous studies have tested the use of minimax robust optimization on phantoms simulating patients with lung tumors [9], and other studies have tested the use of robust optimization on the ITV, replacing the PTV by a robust optimization [10, 11]. To our knowledge, it has not been tested if both the ITV and PTV can be replaced by robust optimization. We hypothesize that robust optimization directly of the GTV could reduce the irradiation of normal tissue without compromising tumor dose.

The aim of this study was to evaluate if planning of photon lung SBRT using robust optimization of the GTV can lead to reductions in normal tissue irradiation, compared to the current standard planning with ITV and PTV, without compromising tumor coverage. To document the clinical utility of the plans, we also report robust evaluations showing target coverage under the influence of breathing motion as observed on 4-Dimensional (4D)-CT and 4D Cone-Beam CT (CBCT).

## Materials and methods

### Patients

This feasibility dose planning study was conducted at the Section for Radiotherapy at the Department of Oncology, Lillebaelt Hospital, University Hospital of Southern Denmark, Vejle, Denmark.

All patients were prospectively included and gave oral and written consent to participate. The study conforms to the Declaration of Helsinki.

Patients with a lung tumor planned for SBRT and complying with the following criteria were offered enrollment in the study. Simultaneous SBRT of one other lesion was allowed.

*Inclusion criteria:*

- Planned SBRT with either 66 Gy in three fractions or 45 Gy in three fractions to a tumor in the lung. Both primary lung cancers and lung metastases from other cancers were allowed.
- Age ≥ 18 years.
- Signed consent to participate.

*Exclusion criteria:*

- Treatment with SBRT of more than two tumors.
- Treatment with SBRT with another dose/fractionation than listed above.
- Treatment of a ground-glass opacity tumor difficult to visualize on CBCT.

### Treatment planning and delineation

Target delineation and treatment planning was performed in RayStation 11B (RaySearch Laboratories, Stockholm, Sweden) using the Collapsed Cone v. 5.4 dose algorithm, with two partial arcs delivering dose through the diseased lung only. All patients received our standard SBRT treatment, which was delivered as Volumetric Modulated Arc Therapy (VMAT) on Elekta linacs equipped with the Agility Multi-Leaf Collimator (MLC), using a 10 Mega-Voltage (MV) Flattening Filter Free (FFF) beam (Elekta AB, Stockholm, Sweden).

All patients went through our Department’s standard PTV-based treatment planning: First, a 4D-CT was performed. The GTV was contoured by an oncologist on the mid-position planning phase. A script using deformable registration was used to propagate the GTV on the other phases.

### Review and scoring of GTV delineations

To ensure that the delineations of GTV met our departmental standards, the GTV on all 10 phases for the first 10 patients were reviewed independently by two experienced lung oncologists and scored on a Likert-scale between 1 and 5 inspired by Palazzo et al. [12]. The definitions for the scores were as follows: 5 – No changes needed – contouring is acceptable; 4 – Only small changes suggested – contouring is acceptable; 3 – Moderate changes needed – contouring is not acceptable; 2 – Large changes needed – contouring is not acceptable; 1 – Almost complete re-contouring necessary – contouring is not acceptable.

### Clinical PTV-based planning strategy

The GTV from all 10 respiratory phases was summed to an ITV before adding a 5 mm isotropic margin to create a PTV. Treatment was planned on the mid-position respiratory phase without density override on the ITV.

All PTV-based plans were optimized in the same way initially, with focus on ensuring coverage of the GTV (95% of prescribed dose) and PTV (67% of prescribed dose). For conformity, a ring Region of Interest (ROI) was created 1–2 cm from the PTV, and a steep dose fall-off function was applied to this ring. A low weight optimization objective was added to keep dose to the thoracic wall below the 35 Gy/45 Gy constraint. We had no limitations for the maximum dose inside the GTV. To prevent high dose outside the tumor, we aimed to keep 100% of the prescribed GTV dose in the ‘PTV outside GTV’ at no more than 5%, but this soft constraint was relaxed if target coverage or Organs At Risk (OAR) constraints were not fulfilled. Only if OAR constraints were violated, organ specific objectives were added to the optimization. A table showing our planning constraints is provided in Supplementary file 1.

### Robust optimization strategy

After each patient had completed treatment based on the PTV-plan, a plan using the robust optimization functionality in RayStation was made. The same delineations of GTV and OARs were used. Instead of creating an ITV and adding margins to form the PTV, a robust optimization objective on the GTV was introduced. A 5 mm isocenter shift was applied to create the 14 robust scenarios from the mid-position CT (six orthogonal and eight diagonal isocenter position shifts of the GTV), and additionally the maximum inhale and exhale phases were included to create a total of 16 scenarios in the robust optimization. The goal for target coverage in the robust optimization was similar to the PTV in the normal planning strategy (67% of prescribed dose covering the GTV with 95% of prescribed dose covering the GTV only in the mid-position phase). All other plan objectives besides target coverage were only applied to the mid-position planning phase, and not robust optimized (to mimic the PTV-based planning strategy). The same ring structure was used as in the PTV-based planning strategy to create conformal plans, and the same purpose could be served by creating a ring structure based directly on the GTV. No objectives were added to limit the high dose region in addition to the dose fall-off on the ring. Supplementary file 2 shows an example of the structures and phases used for plan optimization.

Following robust optimization, the plans were evaluated in all the same scenarios used during optimization (5 mm isocenter shift + extreme respiratory phases). If GTV V67% > 98% was fulfilled in all scenarios, the plan was considered robust towards motion and setup uncertainties. There was no deformation or recalculation to breathing phases.

All robust optimized plans were drafted using Python scripts (available in Supplementary file 3). The scripts determined the mid-position phase and the maximum inspiratory and expiratory phases, by determining the two phases with GTV centers farthest away from the mid-position in opposite directions. The scripts executed the robust optimization and finally made the robust plan evaluation with 5 mm isocenter shift. Manual optimization was subsequently only performed if the clinical goals were not fulfilled. Plan data was extracted by script from RayStation in comma-separated files.

### Tumor motion and robust evaluation

Tumor motion was extracted from the 4D CT scan, as well as from 4D CBCTs from the treatment course to evaluate the actual motion of each tumor. The motion amplitude on 4D CT was extracted based on the GTV midpoint from the delineations on all phases. On 4D CBCT, the tumor motion amplitude was extracted as the largest amplitude observed during pre- and mid-treatment 4D CBCT using automatic match in the Elekta XVI 5.0 software (Elekta AB, Stockholm, Sweden).

This motion data was used for robust plan evaluation of both the PTV-based plan and the plan using robust optimization to elucidate if the plans ensured adequate tumor coverage given the observed tumor motion. This means the largest amplitudes were used for the isocenter shifts instead of the 5 mm used for the planning with robust optimization.

### Statistics

Final data analysis was done in STATA ver. 18 (StataCorp, College Station, TX) and MATLAB ver. 2015A (MathWorks, Natick, MA). Continuous variables were reported as means with standard deviation, if normally distributed (tested with visual inspection of QQ-plots), and as median with range and/or inter-quartile range (IQR) if non-normally distributed. Medians were compared using the Wilcoxon signed rank test of equal ranks.

This was a feasibility study with a new planning technique not used at our Department before. Actual sample size calculations could therefore not be made, as we had no means of anticipating what changes the robust planning strategy would produce. We expected that approximately 40 patients would be sufficient to reveal meaningful differences between the two treatment planning methods.

## Results

### Scoring of the GTV delineations for the first 10 patients and review of the PTV-based plans

The GTV delineations were acceptable, with a mean score of 4.7 (scoring shown in Supplementary file 4).

Every PTV-based patient plan was reviewed to assess, whether the optimization had been satisfactory, or if the plan could have been optimized further. We identified a few plans that we believe could have been optimized a little further and gained slightly better tumor coverage (2 plans), or less dose to the Thoracic Wall (3 plans). All these deviations were deemed minor and without clinical significance by a senior oncologist, so the original plans were used for the comparisons.

### Patient characteristics

Forty patients were included between September 1, 2022 and August 31, 2023.

The patient characteristics are shown in Table 1.

**Table 1:** Patient and treatment characteristics.

Total number of patients	39 (100%)
Age, median (range and IQR)	74 years (57–84 years – IQR 10 years)
Gender, *n* (%)	
Female	16 (41%)
Male	23 (59%)
Performance status, *n* (%)	
1	26 (67%)
2	11 (28%)
3	2 (5%)
SBRT dose prescription, *n* (%)	
66 Gray in 3 fractions	32 (82%)
45 Gray in 3 fractions	7 (18%)
GTV, median (range and IQR)	3.19 ccm (0.3–23.0 ccm – IQR 4.17 ccm)
PTV, median (range and IQR)	17.6 ccm (5.1–67.7 ccm – IQR 16.4 ccm)
Minimum distance from GTV to thoracic wall, median (range and IQR)	0.5 cm (0.1–2.9 cm – IQR 0.9 cm)

GTV: Gross Tumor Volume; IQR: Interquartile Range; PTV: Planning Target Volume.

During data analysis, it turned out that the planning scan of one patient was of poor quality with artifacts in the treatment region, probably due to irregular breathing. It was deemed impossible to make proper comparisons of different treatment plans due to these artifacts, and the patient was excluded from the cohort, so 39 patients were included in the final analyses.

### Plan comparisons

**Figure 1:**
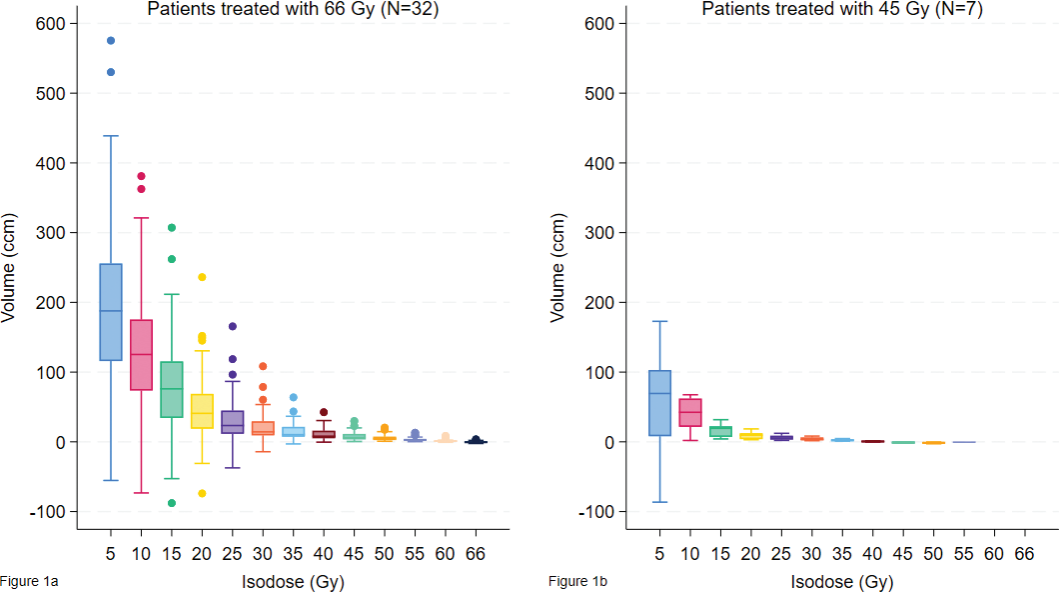
(A and B) Box plots showing the reduction in isodose volumes with the robust optimized plans compared to the normal plans for the patients receiving 66 Gy (Figure 1A) and the patients receiving 45 Gy (Figure 1B).

The plan analyses revealed that it was possible to reduce the irradiation of normal tissue. For the patients treated with 66 Gy, all isodose volumes below 66 Gy and all OAR doses except Bronchus_PRV were statistically significantly lower when using robust optimization instead of PTV-based planning (*p* < 0.05 in all instances, see Supplementary file 5). There were only seven patients treated with 45 Gy, but we did find statistically significant reductions of most isodose volumes and a few OAR doses for these patients as well, as seen in Supplementary file 5. The reduction was largest for the low dose volumes, as seen in Figure 1A and B. The slight increase in the 66 Gy/45 Gy isodose volumes with robust optimization is linked to higher GTV mean dose seen in the robust optimized plans, see Table 2.

**Table 2:** Changes in doses to GTV and thoracic wall accumulated from all 10 respiratory phases and/or mid-position phase between the two planning strategies and differences in plan Monitor Units.

Change in plan parameter (Robust – PTV-based)	Value (median, IQR)	*p*-value (Signed Rank test)
Accumulated Mean GTV dose	4.46 Gy (4.5)	< 0.001
Mid-position phase mean GTV dose	5.92 Gy (4.57)	< 0.001
Accumulated GTV V42.75 Gy	0 ccm (0.0)	0.78
Mid-position phase GTV V42.75 Gy	0 ccm (0.02)	0.16
Accumulated GTV V62.7 Gy	0.01 ccm (2.17)	0.43
Mid-position phase GTV V62.7 Gy	0 ccm (1.02)	0.84
Mid-position phase Thoracic Wall D0.05 ccm	-0.73 Gy (3.55)	0.007
Mid-position phase Thoracic Wall D1 ccm	-2.44 Gy (2.32)	< 0.001
Monitor units	102.6 MU (785.6)	0.39

GTV: Gross Tumor Volume; IQR: Interquartile Range; MU: Monitor Units; PTV: Planning Target Volume.

**Figure 2:**
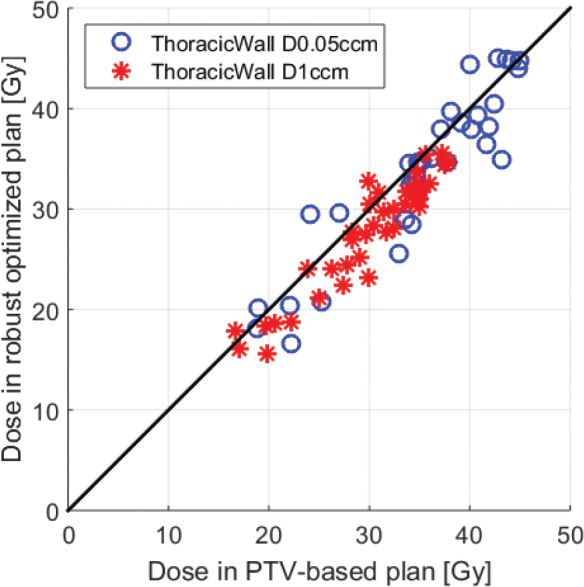
Plot of Thoracic Wall D0.05ccm and D1ccm for the robust optimized plans (y-axis) versus the PTV-based plan (x-axis). The black line indicates identical doses in the two plans.

Figure 2 shows that it was possible in most instances to reduce the dose to the thoracic wall for both D0.05ccm and D1ccm with the robust optimized plans.

**Figure 3:**
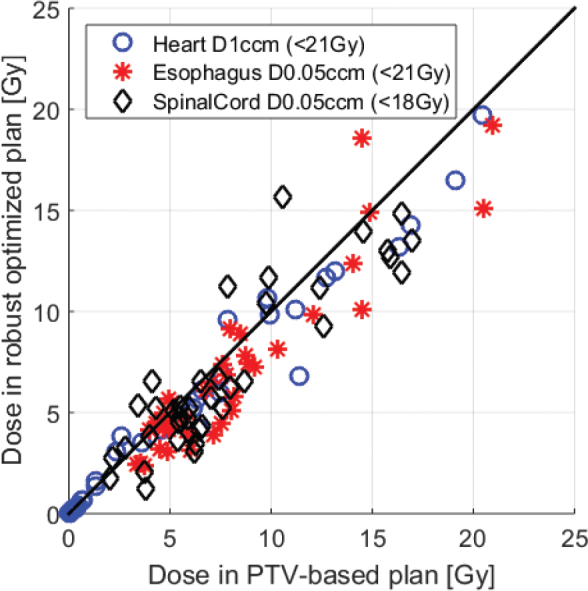
Plot of Heart D1cmm, Esophagus D0.05ccm, and SpinalCord D0.05ccm for the robust optimized plans (y-axis) versus the PTV-based plan (x-axis). The black line indicates identical doses in the two plans. Because of the small doses in most cases, the differences might be random or related to the reductions in isodose volumes.

Figure 3 shows the obtained doses for heart, esophagus, and spinal cord with a reduction in the robust optimized plans in most instances.

Following the scripted robust optimization and evaluation, 20 of the 39 plans complied with all our clinical plan constraints. The main deviations in the remaining plans was a dose exceeding the allowed in the thoracic wall (18 out of 19). Three plans had a too low GTV dose and seven plans had too high dose to ribs, spinal cord, esophagus, bronchus, or brachial plexus.

Following manual optimization, 36 of the 39 plans made with robust optimization complied with our constraints in all 14 scenarios using 5-mm isocenter shift and in all respiratory phases. Three of the 39 plans could only be made robust in 13 of 14 scenarios, but they were still robust in all respiratory phases. The limiting factor for these three plans was maximum dose to the thoracic wall, where the one scenario moving the isocenter closer to the thoracic wall (i.e. the ROIs became overlapping) could not simultaneously keep the thoracic wall D0.05 ccm < 45 Gy and the GTV V42.75 Gy > 99.9%.

Tumor dose was not only preserved, but actually increased with the robust optimized plans, see Table 2. There was a slight increase in median monitor units (MU) in the robust plans, though not statistically significant.

### Observed tumor motion

Tumor motion was generally smaller on the 4D-CT planning scan compared to the CBCT’s performed before delivery of each treatment fraction, see Table 3.

**Table 3:** Median peak-to-peak amplitudes on the 4D-CT planning scan and the CBCTs during treatment.

Peak-to-peak amplitude – Median (range)	4D-CT planning scan	CBCTs during treatment
Left-Right	0.14 cm (0.015–0.68 cm)	0.25 cm (0.080–1.1 cm)
Anterior–Posterior	0.26 cm (0.061–1.1 cm)	0.44 cm (0.10–1.4 cm)
Cranial–Caudal	0.35 cm (0.040–1.9 cm)	0.58 cm (0.080–3.3 cm)
Number of patients with amplitudes of > 1.0 cm	4D-CT planning scan	CBCTs during treatment
Left-Right	0 (0%)	1 (2.6%)
Anterior–Posterior	2 (5.1%)	4 (10.3%)
Cranial–Caudal	4 (10.3%)	11 (28.2%)

4D-CT: 4-Dimensional Computed Tomography; CBCT: Cone-Beam Computed Tomography.

### Robust plan evaluations with the observed tumor motion

**Figure 4:**
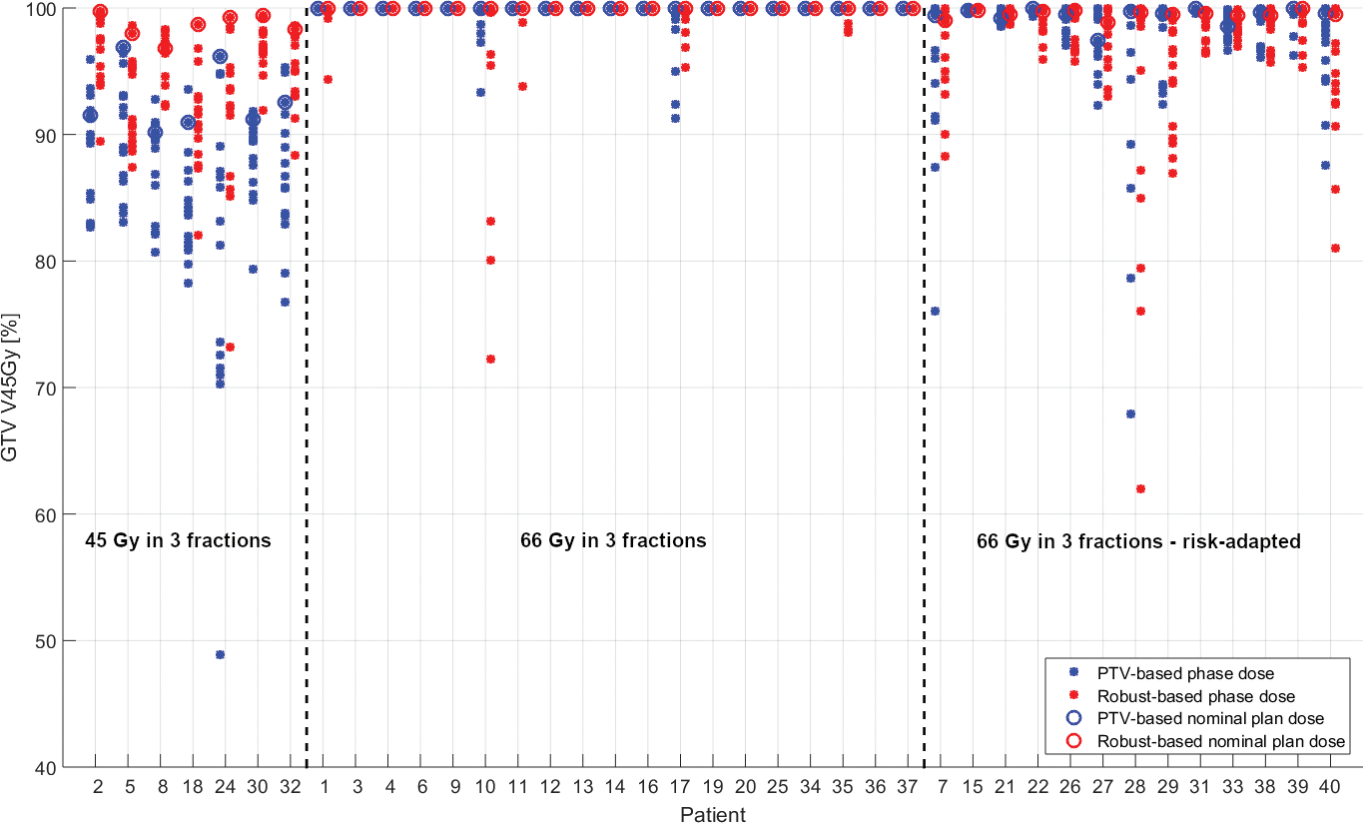
GTV V45Gy for each of the 14 scenarios evaluated with observed tumor motion for both plans from every patient grouped by prescription and whether the tumor was close to the thoracic wall (‘Risk-adapted’). For each patient there are 2 × 14 dots. The figure is grouped so the plots for the seven patients receiving 45 Gy in three fractions are shown to the left.

The observed maximum amplitudes for the six nominal directions (Ant, Post, Sup, Inf, Left, Right) for each patient were then used for robust plan evaluation with the observed tumor motion of both the PTV-based and the robust optimized plan. The differences in coverage of GTV V45Gy for all 14 scenarios for both PTV-based and robust optimized plans are shown in Figure 4. In Figure 4, the patients receiving 66 Gy in three fractions are grouped according to proximity to the thoracic wall, which explains the many points below 98% in the risk-adapted right section of the graph. We report V45Gy, as this was a relevant dose level for all patients (67% of the prescription dose for the 66 Gy patients and 100% for the 45 Gy patients). Figure 4 shows that the coverage of the two planning techniques is at least equally good, but with a tendency of better coverage with the robust optimized approach.

## Discussion

This study documents that statistically significant reductions in irradiated volumes can be obtained with robust optimization in pulmonary photon SBRT, without compromising tumor coverage, even when applying the actual tumor motion seen for each patient in the robust evaluation. For some patients, the PTV-based plan had a slightly better tumor coverage, while for other patients the robust optimized plan had better tumor coverage. We have not been able to identify reasons for this dichotomy.

The reductions in irradiated volume are largest for the low-dose volumes and the crucial question is, whether these statistically significant reductions can also be regarded as clinically significant? As the risk of a range of side effects increases with the total volume or dose of irradiated normal tissue [13], one could argue that every possible reduction of the irradiated tissue is worth obtaining to reduce the risk of side effects as much as possible. On the other hand, in the everyday clinical practice, there is only so much time available to work with each plan, and improvements must either be of a certain magnitude or not cause more than a few minutes of extra work to be feasible to implement.

We experienced widely different time spent on creating the two plans. It took an estimated factor five longer to prepare the robust plans compared to the average duration of around 15 min for a normal plan. A part of the prolonged planning time was due to the robust optimization requiring more computing power, but the process also required more labor from the medical physicist preparing the plan. With the use of the different scripts, many parts of the robust optimization were automated, but the medical physicist had to interact with the TPS at different time points during the execution of the scripts. With further programming, some of these interactions might be removed (i.e. entering the tumor location and dose prescribed). With advances in computing power, the excess planning time used might be reduced to some extent.

As the robust optimization was only focused on obtaining adequate GTV dose and complying with the thoracic wall constraints, it was a bit surprising to see that the GTV mean dose was increased by almost 4,5 Gy median. The difference is likely caused by a soft constraint on the PTV-based plan, where 100% dose is limited to 5% of the volume between the GTV and PTV – a constraint which is not found in the robust optimized plans (since no PTV is present). As it has been suggested that obtaining a hotspot inside the SBRT target [14], or obtaining a BED > 130 Gy might carry better chances of local tumor control [15], this increase is likely of benefit to the patients. A similar increase in GTV dose might be obtained in the PTV-based plans, if that soft constraint is removed.

The increased tumor motion on the CBCT scans can have several explanations: An elastic belt measuring breathing motion is placed on the abdomen during the 4D-CT scan, which might unknowingly have restricted the patients’ breathing. The patients might also have felt tenser during the 4D-CT scan, as it is often performed on the same day as their initial consultation at the Radiotherapy section, and that might have caused their breathing to be more shallow. Finally, the difference in image acquisition and reconstruction between 4D-CT and 4D-CBCT may also cause different appearance of the tumor motion.

Though the tumor motion seen on CBCT scans had larger amplitudes than the 4D-CT planning scans, which differs somewhat from another study comparing the two [16], it might still not reflect the actual intrafraction tumor motion during treatment [17]. Interplay effects of the delivered VMAT treatment with the moving tumor cannot be estimated in the current approach, but requires tumor tracking and segment-based dose calculation on 4D images acquired during treatment.

Since the plans with robust optimization took substantially longer to create, we believe that this strategy is too time consuming to be used in the daily SBRT treatment planning for all patients. There may however be certain situations, where the reductions obtainable will be worth the extra time. We had two patients with apical tumors located close to the brachial plexus, and the dose reduction to this OAR (median reduction 1.63 Gy) might lower the risk of severe neuropathy for this subgroup of patients. It will take further studies with more patients with apical tumors to determine if this is indeed the case.

We believe that our results are generalizable to other institutions as well. Most TPS’ now offer robust optimization planning capabilities, and if another GTV to PTV margin is used, it can still be applied to the isocenter shifts of the robust optimization.

Strengths of this study include the rigorous and detailed planning strategy of both approaches and the validations of GTV delineation and normal plans before the comparisons.

Limitations of this study include the lack of outcome data. The pre-selected number of patients may have imposed some degree of uncertainty. We were not able to calculate the actual delivered dose to the tumor, which should be the ultimate deciding factor in choosing the optimal treatment planning strategy.

## Conclusion

This study shows statistically significant reductions in irradiated volumes without compromising tumor coverage when using robust optimization for the planning of lung tumor SBRT with photons compared to our standard PTV-based planning strategy. The robust optimized plans took substantially longer time to create, so we do not expect this strategy to be relevant for all future lung SBRT patients. We believe that the use may be relevant in selected cases, for instance when treating apical tumors close to the brachial plexus, where you want to reduce the OAR dose as much as possible. More work is required to fully determine the potential of this technique in photon lung SBRT planning.

## Author contributions

Conceptualization: T.L.F., R.S.T., C.K., T.S.H., and T.F.H. Investigation: T.L.F., R.S.T., C.K., and T.S.H. Methodology: T.L.F., R.S.T., C.K., and T.S.H. Project administration: T.L.F., R.S.T., C.K., and T.S.H. Robust plan creation using scripts: T.L.F. Robust plan manual optimization: R.S.T. Validation of GTV delineations: C.K. and T.S.H. Validation of normal plans: R.S.T. and C.K.. Validation of other data and results: R.S.T., T.F.H., C.K., and T.S.H. Supervision: R.S.T., T.F.H., C.K., and T.S.H. Statistics: T.L.F. and R.S.T. Visualization: T.L.F., R.S.T., C.K., and T.S.H. Writing – original draft: T.L.F. and R.S.T. Writing – review & editing: T.L.F., R.S.T., C.K., T.S.H. and T.F.H.

All authors have read and agreed to the published version of the manuscript.

## Supplementary Material

Robust optimization of the Gross Tumor Volume compared to conventional Planning Target Volume-based planning in photon Stereotactic Body Radiation Therapy of lung tumors

## Data Availability

The data material related to this article can be provided after written request to the corresponding author.
