# Supplementary material for: Robust optimization of the Gross Tumor Volume compared to conventional Planning Target Volume-based planning in photon Stereotactic Body Radiation Therapy of lung tumors
Source: Acta Oncol. 2024 Jun 20;63:40049. doi: 10.2340/1651-226X.2024.40049 (PMC11332535; doi:10.2340/1651-226X.2024.40049)
Supplement: Robust optimization of the Gross Tumor Volume compared to conventional Planning Target Volume-based planning in photon Stereotactic Body Radiation Therapy of lung tumors [file AO-63-40049-s1.pdf]

Supplementary material has been published as submitted. It has not been copyedited, or typeset by Acta Oncologica

Supplement 1 – Planning constraints. All constraints are mandatory unless otherwise specified.

	**66 Gy / 3 fr.**	**45 Gy / 3 fr.**
**Targets**		
GTV	$D_{0.1ccm} \geq 66$ Gy<br>$V_{62.7Gy} \geq 95$ % (mandatory),<br>$V_{62.7Gy} \geq 99$ % (optimal)	$D_{0.1ccm} \geq 45$ Gy<br>$V_{42.75Gy} \geq 99.9$ %
PTV	$V_{45Gy} \geq 98$ % (mandatory),<br>$V_{45Gy} \geq 99$ % (optimal)	$V_{30Gy} \geq 98$ % (mandatory),<br>$V_{30Gy} \geq 99$ % (optimal)
**OAR**		
SpinalCord	$D_{0.05ccm} < 18$ Gy	
Lungs	$V_{13Gy} < 30$ %	
Esophagus	$D_{0.05ccm} < 21$ Gy	
Heart	$D_{1.0ccm} < 21$ Gy	
Trachea	$D_{0.05ccm} < 21$ Gy	
Bronchus	$D_{0.05ccm} < 30$ Gy	
Bronchus_PRV	$D_{0.05ccm} < 30$ Gy (optimal)	
Ribs	$D_{0.05ccm} < 45$ Gy	
Aorta	$D_{0.05ccm} < 35$ Gy	
ThoracicWall	$D_{0.05ccm} < 45$ Gy (mandatory), $D_{0.05ccm} < 35$ Gy (optimal)	
Skin	$D_{1.0ccm} < 25$ Gy	
BrachialPlexus	$D_{0.05ccm} < 28$ Gy	
External	Hotspots > prescribed dose (66/45 Gy) outside the GTV are avoided.<br>In practice (PTV-GTV) $V_{100\%} \leq 5$ % is accepted.	

Explanation: $D_{0.1ccm} \geq 66$ Gy: Dose to 0.1 cubic centimeter receiving more than or equal to 66 Gray.

PRV: Planning Risk Volume.

Priorities:

1. There must be areas inside the GTV receiving at least the prescribed dose (i.e. $D_{0.1ccm} \geq$ prescribed dose).
2. The PTV must be surrounded by approx. 2/3 of the prescribed dose (i.e. $V_{45Gy} \geq 98\%$ for 66 Gy/3 fr.)
3. The GTV $V_{95\%}$ is increased as much as possible without increasing the PTV isodose curve
4. All OAR constraints must be complied with, otherwise consider another fractionation.

Supplement 2 – Figure showing the structures used for optimization

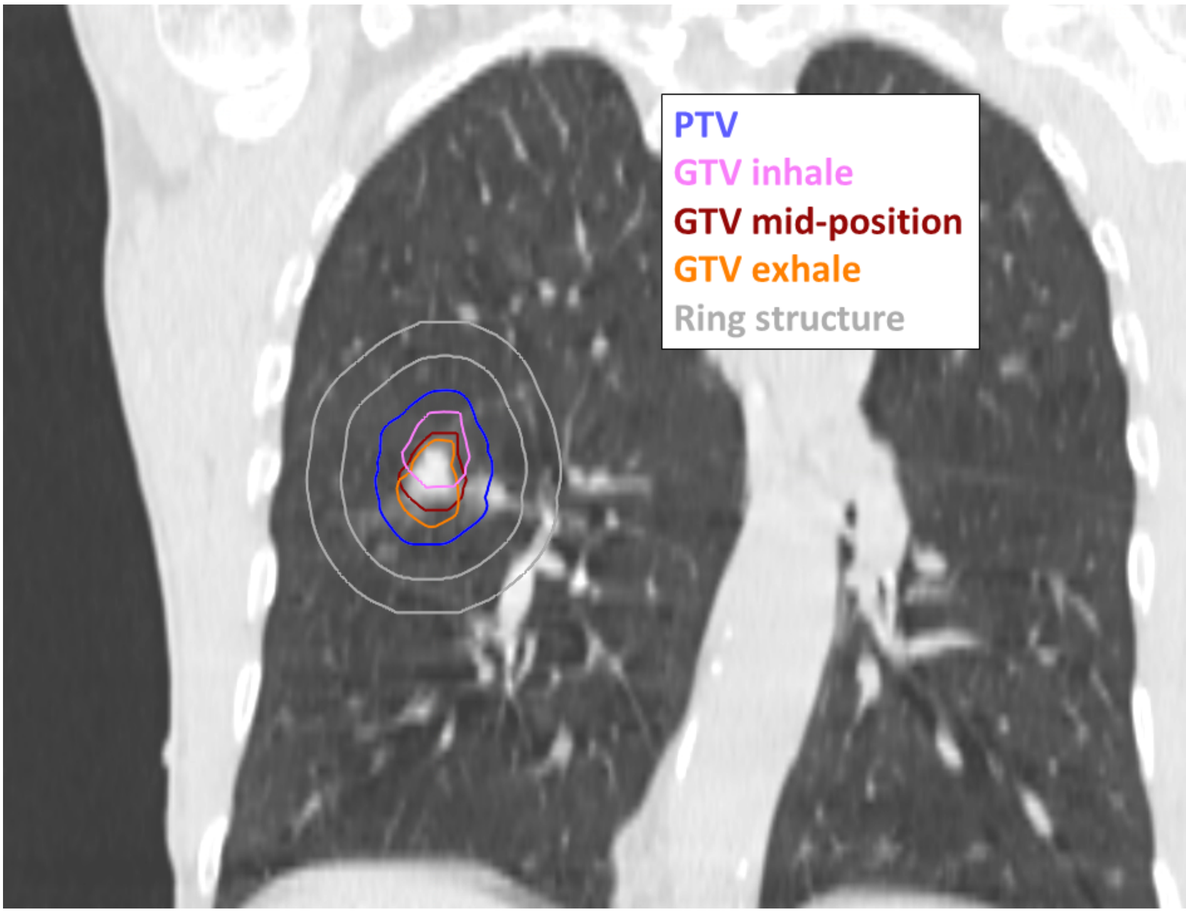

Supplement 3 – Scripts used for planning

Script 1: Determine GTV position on phases of 4D-CT:

```
import math
from connect import *
import time
import sys
import re
import clr
import numpy as np

from tkinter import *
import tkinter.messagebox

FilePath =

# Set current handles
Patient = get_current('Patient')
cs = get_current('Case')
exam = get_current('Examination')
pm = cs.PatientModel
rois = pm.StructureSets[exam.Name]
#Plan = get_current('Plan')
#beam_set = get_current('BeamSet')

###### Identify examination group which the current examination belongs to: ######
### list examination groups
eGroupLst = []
for i in cs.ExaminationGroups:
    eGroupLst.extend([i.Name])

### determine which examination group has the current examination in it
for i in eGroupLst:
    for j in cs.ExaminationGroups[i].Items:
        if j.Examination.Name == exam.Name:
            eGroupName = i
eGroup = cs.ExaminationGroups[eGroupName]

### Build list of CTs in 4D group
dlst = []
dlst_total = []
for i in eGroup.Items:
    dlst_total.extend([i.Examination.Name]) # list of all CTs in the 4D group
    if i.Examination.Name != exam.Name:
        dlst.extend({i.Examination.Name}) # list of CTs in the 4D group, without the primary scan.

x = []
y = []
z = []
for exm in dlst_total:
    temp = pm.StructureSets[exm].RoiGeometries['GTVp'].GetCenterOfRoi()
    x.extend([temp.x])
    y.extend([temp.y])
    z.extend([temp.z])


print('Motion amplitude in x is '+str(np.max(x)-np.min(x)))
print('Motion amplitude in y is '+str(np.max(y)-np.min(y)))
```

```
print('Motion amplitude in z is '+str(np.max(z)-np.min(z)))

xm = np.mean(x)
ym = np.mean(y)
zm = np.mean(z)

xdist = []
ydist = []
zdist = []
rms = []
for i in range(len(x)):
   xdist.extend([x[i]-xm])
   ydist.extend([y[i]-ym])
   zdist.extend([z[i]-zm])

ind = np.argmin(np.sqrt(np.square(x-xm) + np.square(y-ym) + np.square(z-zm)))

dist_from_mean = np.sqrt(np.square(x-xm) + np.square(y-ym) + np.square(z-zm))

for i in range(len(x)):
                        print('GTV center on ' + dlst_total[i] + ' is ' + str(dist_from_mean[i]) + ' cm from mean position')

print('The 4D phase with GTV center closest to the mean position is '+dlst_total[ind])
```

Script 2: Create robust plan:

```
from connect import *
from tkinter import *
import tkinter.messagebox
import os
import io


def GetHandles():
    '''Function to return major handles in a list
    @return: list containing Raystation handle objects: case, patient, patient database, patient model, examination, structure set (Rois).
    '''
    case        = get_current('Case')
    patient      = get_current('Patient')
    patientDB    = get_current('PatientDB')
    patientModel = case.PatientModel
    examination  = get_current('Examination')
    structureSet = case.PatientModel.StructureSets[examination.Name]
    return [case, patient, patientDB, patientModel, examination, structureSet]


def SetVmatOptimizationParam(optParam, iterations = 40, tolerance = 1E-06, useLeafTravelDistance = 'True', maxLeafTravelDistance = 0.80, numberOfArcs = 2, gantrySpacing = 4, maxDeliveryTime = 90):
    try:
        optParam.Algorithm.MaxNumberOfIterations  = iterations # 40 default
        optParam.Algorithm.OptimalityTolerance    = tolerance  # 1E-06 default
        optParam.DoseCalculation.ComputeFinalDose = 'True'
        optParam.DoseCalculation.ComputeIntermediateDose = 'True'
        optParam.DoseCalculation.IterationsInPreparationsPhase = 7
        optParam.TreatmentSetupSettings[0].SegmentConversion.ArcConversionProperties.UseMaxLeafTravelDistancePerDegree = useLeafTravelDistance # 'True' default
        optParam.TreatmentSetupSettings[0].SegmentConversion.ArcConversionProperties.MaxLeafTravelDistancePerDegree    = maxLeafTravelDistance # 0.80 default
    except Exception:
        raise Exception('Error - could not set VMAT optimization parameters by script.')
    try:
        optParam.TreatmentSetupSettings[0].BeamSettings[0].ArcConversionPropertiesPerBeam.NumberOfArcs = numberOfArcs # 2 default
    except Exception:
        raise Exception('Error - could not set VMAT the number of arcs by script.')

    try:
        if numberOfArcs == 2:
            dualArcs = True
        else:
            dualArcs = False
        for beams in optParam.TreatmentSetupSettings[0].BeamSettings:
            beams.ArcConversionPropertiesPerBeam.EditArcBasedBeamOptimizationSettings(CreateDualArcs=dualArcs, FinalGantrySpacing=gantrySpacing, MaxArcDeliveryTime=maxDeliveryTime, BurstGantrySpacing=None, MaxArcMU=None)
    except Exception:
        raise Exception('Error - could not set gantry spacing and max delivery time by script')

def SelectDoseFracLat(GTVs):
    '''GUI to select lung dose/fractionation and laterality
    @return: returns Dose, Fractions, Laterality and Primary GTV
    '''
    master = Tk()

    # Heading:
```

```
    Label(master, text='   Lung SBRT planning script invoked   ',font=('Arial',14)).pack()
    Label(master, text='').pack() # add empty line

    # Buttons to select dose/fractionation:
    Label(master, text='Please select dose and fractionation:').pack()
    DoseFrac = StringVar()
    Radiobutton(master, text='66Gy/3F', variable=DoseFrac, value='66,3', indicatoron=0).pack()
    Radiobutton(master, text='45Gy/3F', variable=DoseFrac, value='45,3', indicatoron=0).pack()
    Label(master, text='').pack() # add empty line

    # Buttons to select laterality:
    Label(master, text='Please select laterality:').pack()
    Side = StringVar()
    Radiobutton(master, text='Left (Gantry 179->340 degrees CounterClockwise)', variable=Side, value='Left', indicatoron=0).pack()
    Radiobutton(master, text='Right (Gantry 181->20 degrees Clockwise)', variable=Side, value='Right', indicatoron=0).pack()
    Label(master, text='').pack() # add empty line

    # Drop down menu to select primary GTV:
    Target = StringVar(master)
    Target.set(GTVs[0]) # Default value
    Label(master,text='Please select primary target:').pack()
    OptionMenu(master,Target,*GTVs).pack()

    Label(master, text='').pack() # add empty line
    Button(master,text='OK',command=master.quit, font=('Arial',14)).pack()

    master.lift() # bring the pop-up window to the front of the screen
    mainloop() # create the window
    master.destroy() # close the window after pressing "OK"

    # Return relevant parameters_
    Dose = int(DoseFrac.get().split(',')[0])*100
    Frac = int(DoseFrac.get().split(',')[1])
    return [Dose, Frac, Side.get(), Target.get()]

# Get major handles
case, patient, patientDB, pm, examination, rois = GetHandles()

# -------------------------------------- Perform checks before planning ----------------------------------

# List all existing ROIs:
ExistingROIs = [rg.OfRoi.Name for rg in rois.RoiGeometries]
# List GTVs excluding MIP and _Total
GTVs = [rg.OfRoi.Name for rg in rois.RoiGeometries if (rg.OfRoi.Name.startswith('GTV') and ("MIP" not in rg.OfRoi.Name) and
('_Total' not in rg.OfRoi.Name) )]

# Get dose, fractionation and laterality:
Dose, Fractions, Laterality, SelectedGTV = SelectDoseFracLat(GTVs)

print('Continuing to add a plan with '+str(Dose/100)+' Gy in '+str(Fractions)+' fractions located '+Laterality+' with '+SelectedGTV+'
as primary (match) target')

SelectedPTV = SelectedGTV.replace('G','P',1)


PTV_GTV = 'NS_'+SelectedPTV+'-'+SelectedGTV
PTVring = 'NS_'+SelectedPTV+'_10-20'
TWnearPTV = 'NS_ThoracicWallNear'+SelectedPTV
```

```
#----------------------------------------------------------- Copy couch and create external on other 4D phases
###### Identify examination group which the current examination belongs to: ######
### list examination groups
eGroupLst = []
for i in case.ExaminationGroups:
   eGroupLst.extend([i.Name])

### determine which examination group has the current examination in it
for i in eGroupLst:
   for j in case.ExaminationGroups[i].Items:
      if j.Examination.Name == examination.Name:
         eGroupName = i
eGroup = case.ExaminationGroups[eGroupName]

### Build list of CTs in 4D group
dlst = []
dlst_total = []
for i in eGroup.Items:
   dlst_total.extend([i.Examination.Name]) # list of all CTs in the 4D group
   if i.Examination.Name != examination.Name:
      dlst.extend({i.Examination.Name}) # list of CTs in the 4D group, without the primary scan.


### Create external on other phases
pm.RegionsOfInterest['External'].CreateExternalGeometries(ReferenceExamination=case.Examinations[dlst[0]],
AdditionalExaminationNames=dlst[1:], ReferenceThresholdLevel=None)


DelineatedROIs = [r for r in pm.StructureSets[examination.Name].RoiGeometries if r.HasContours()]
Couch = [r.OfRoi.Name for r in DelineatedROIs if r.OfRoi.Type=='Support']

pm.CopyRoiGeometries(
      SourceExamination=examination,
      TargetExaminationNames=dlst,
      RoiNames=Couch,
      ImageRegistrationNames=[],
      TargetExaminationNamesToSkipAddedReg=dlst)


# --------------------------------------- Add plan ----------------------------------
planPre = 'Robust_'
planName = planPre+str(int(Dose/100))+'_'+str(Fractions)

with CompositeAction(str('Adding plan with name '+planName)):
   plan = case.AddNewPlan(PlanName=planName, Comment='Pair-optimized dual arc lung VMAT plan',
ExaminationName=examination.Name)
   beamSet = plan.AddNewBeamSet(
            Name=planName,
            ExaminationName=examination.Name,
            MachineName='ElektaAgility_v1',
            Modality='Photons',
            TreatmentTechnique='VMAT',
            PatientPosition='HeadFirstSupine',
            NumberOfFractions=Fractions,
            CreateSetupBeams=False)
   beamSet.SetDefaultDoseGrid(VoxelSize={'x': 0.25, 'y': 0.25, 'z': 0.25})
patient.Save()
plan.SetCurrent()


# Set gantry angles depending on laterality:
```

```
print(Laterality)
if Laterality == 'Left':
   VmatGantryStart = 179
   VmatGantryStop = 340
   Rotation = 'CounterClockwise'
elif Laterality == 'Right':
   VmatGantryStart = 181
   VmatGantryStop = 20
   Rotation = 'Clockwise'
else:
   raise Exception('Laterality not defined')

with CompositeAction('Create arc beam'):
   # Set plan isocenter to center of GTV_Total
   isocenter = rois.RoiGeometries[SelectedGTV].GetCenterOfRoi()
   isodata = beamSet.CreateDefaultIsocenterData(Position={'x':isocenter.x, 'y':isocenter.y, 'z':isocenter.z})
   beamSet.CreateArcBeam(
               Name='A1',
               Description='A1',
               BeamQualityId='10 FFF',
               CouchRotationAngle=0,
               CouchPitchAngle=0,
               CouchRollAngle=0,
               GantryAngle=VmatGantryStart,
               ArcStopGantryAngle=VmatGantryStop,
               ArcRotationDirection=Rotation,
               CollimatorAngle=355,
               IsocenterData=isodata)

   if Fractions != 10:
      beamSet.CreateArcBeam(
               Name='A2',
               Description='A2',
               BeamQualityId='10 FFF',
               CouchRotationAngle=0,
               CouchPitchAngle=0,
               CouchRollAngle=0,
               GantryAngle=VmatGantryStart,
               ArcStopGantryAngle=0,
               ArcRotationDirection=Rotation,
               CollimatorAngle=355,
               IsocenterData=isodata)


with CompositeAction('Set optimization parameters'):
   SetVmatOptimizationParam(plan.PlanOptimizations[0].OptimizationParameters, iterations=30, gantrySpacing=2,
numberOfArcs=1, maxDeliveryTime=120, maxLeafTravelDistance=0.4)


# --------------------------------------- Add clinical goals ----------------------------------

defaultScriptingDir, defaultCGdir, defaultOptDir = GetDefaultScriptingDir()

CGfilename = 'SBRTClinicalGoals.csv'
try:
   CGdict = ImportCSVToListOfDicts(os.path.join('.',CGfilename), ';')
   AddClinicalGoalsFromDict(CGdict)
except: # open the file dialog to choose the most appropriate clinical goals file
   try: exec(io.open('ClinicalGoalsFromCSV.py','r').read())
   except: print('No clinical goals added')
```

```
with CompositeAction('Removing MIP and _Total clinical goals'):
    ExcludeROIs = ['*MIP*','*_Total']
    DeleteClinicalGoalsFromList(ExcludeROIs)

# --------------------------------------- Optimization ----------------------------------

# Add optimization objectives - these are based on selected ROIs, and hence not loaded from templates:

if Dose == 6600: PTVDose = 4600
elif Dose == 5000: PTVDose = 3600
else: PTVDose = 3100

OptObj = [{'R': SelectedGTV, 'F': 'MinDose', 'D': Dose, 'W': 100, 'Robust': False},
        {'R': SelectedGTV, 'F': 'MinDose', 'D': PTVDose, 'W': 100, 'Robust': True},
        {'R': PTVring, 'F': 'DoseFallOff', 'HD': Dose, 'LD': 1000, 'Dist': 0.5, 'W': 1, 'Robust': False},
        {'R': TWnearPTV, 'F': 'DoseFallOff', 'HD': 4500, 'LD': 1000, 'Dist': 1, 'W': 1, 'Robust': False},
        {'R': TWnearPTV, 'F': 'MaxDose', 'D': 3400, 'W': 250, 'Robust': False}]

for n, obj in enumerate(OptObj):
    plan.PlanOptimizations[0].AddOptimizationFunction(
        FunctionType = obj['F'],
        RoiName = obj['R'],
        IsConstraint = False,
        RestrictAllBeamsIndividually = False,
        RestrictToBeam = None,
        IsRobust = obj['Robust'],
        RestrictToBeamSet = None,
        UseRbeDose = False
        )

    tempObj = plan.PlanOptimizations[0].Objective.ConstituentFunctions[n].DoseFunctionParameters
    if 'D' in obj:
        try: tempObj.DoseLevel = obj['D']
        except: pass
    if 'V' in obj:
        try: tempObj.PercentVolume = obj['V']
        except: pass
    if 'HD' in obj:
        try:
            tempObj.HighDoseLevel = obj['HD']
            tempObj.LowDoseLevel = obj['LD']
            tempObj.LowDoseDistance = obj['Dist']
        except: pass
    tempObj.Weight = obj['W']

# Set 0.5 cm robust
plan.PlanOptimizations[0].OptimizationParameters.SaveRobustnessParameters(PositionUncertaintyAnterior=0.5,
PositionUncertaintyPosterior=0.5, PositionUncertaintySuperior=0.5, PositionUncertaintyInferior=0.5, PositionUncertaintyLeft=0.5,
PositionUncertaintyRight=0.5, DensityUncertainty=0, PositionUncertaintySetting="Universal", IndependentLeftRight=True,
IndependentAnteriorPosterior=True, IndependentSuperiorInferior=True, ComputeExactScenarioDoses=False,
NamesOfNonPlanningExaminations=[], PatientGeometryUncertaintyType="PerTreatmentCourse",
PositionUncertaintyType="PerTreatmentCourse", TreatmentCourseScenariosFactor=1000)

# --------------------------------------- Script completed ----------------------------------
tkinter.messagebox.showinfo(title='Script completed',message='Plan created. Please select appropriate 4D image sets to include in
robust optimization, add these and run optimization.')
```

Script 3: Optimize 3 times

```
from datetime import datetime
from connect import *
## Script execution time
scriptStartTime = datetime.now()

plan = get_current('Plan')

# Run 3 optimizations:
for w in range(3):
    plan.PlanOptimizations[0].RunOptimization()

print('\n--- Script Execution Time {} seconds ---\n'.format(datetime.now()-scriptStartTime))
```

Script 4: Calculate robust evaluation doses:

```
from connect import *
beam_set = get_current("BeamSet")

retval_0 = beam_set.CreateRadiationSetScenarioGroup(Name="RobustEval_5mm", UseIsotropicPositionUncertainty=False,
PositionUncertaintySuperior=0.5, PositionUncertaintyInferior=0.5, PositionUncertaintyPosterior=0.5,
PositionUncertaintyAnterior=0.5, PositionUncertaintyLeft=0.5, PositionUncertaintyRight=0.5,
PositionUncertaintyFormation="AxesAndDiagonalEndPoints", PositionUncertaintyList=None, DensityUncertaintyPercent=0,
NumberOfDensityDiscretizationPoints=2, ComputeScenarioDosesAfterGroupCreation=False)

retval_0.ComputeScenarioGroupDoseValues()
```

Supplement 4 - Scoring of GTV delineations

Patient number:	Score by reviewer 1:	Score by reviewer 2:
1	4	5
2	5	5
3	5	5
4	5	5
5	4	5
6	4	5
7	4	5
8	4	5
9	5	5
10	4	5
Average score:	4.4	5.0

Average score combined = 4.7

Supplement 5 – Changes in isodose volumes, OAR doses and corresponding p-values. Patient numbers are 32 (66 Gy patients) or 7 (45 Gy patients) unless otherwise specified in the table.

	**66 Gy patients (N=32)**		**45 Gy patients (N=7)**	
**Isodose or OAR:**	**Median change in volume or dose (PTV-based - robust)**	**p-value: (Signed Rank)**	**Median change in volume or dose (PTV-based - robust)**	**p-value: (Signed Rank)**
5 Gy isodose volume	187.7 ccm	< 0.001	69.4 ccm	= 0.16
10 Gy isodose volume	125.2 ccm	< 0.001	42.3 ccm	= 0.02
15 Gy isodose volume	76.1 ccm	< 0.001	19.4 ccm	= 0.02
20 Gy isodose volume	40.8 ccm	< 0.001	9.6 ccm	= 0.02
25 Gy isodose volume	23.4 ccm	< 0.001	5.5 ccm	= 0.02
30 Gy isodose volume	14.3 ccm	< 0.001	3.1 ccm	= 0.02
35 Gy isodose volume	10.3 ccm	< 0.001	1.6 ccm	= 0.02
40 Gy isodose volume	8.1 ccm	< 0.001	0.3 ccm	= 0.08
45 Gy isodose volume	6.3 ccm	< 0.001	- 1.1 ccm	= 0.02
50 Gy isodose volume	4.6 ccm	< 0.001	- 1.2 ccm	= 0.02
55 Gy isodose volume	2.8 ccm	< 0.001	- 0.5 ccm (n = 1)	= 1 (n = 1)
60 Gy isodose volume	0.9 ccm	< 0.001	N/A	N/A
66 Gy isodose volume	- 0.6 ccm	= 0.07	N/A	N/A
Spinal Cord D0.05ccm	1.5 Gy	= 0.003	0.2 Gy	= 1
Lungs V13Gy	0.6 ccm	< 0.001	0.2 ccm	= 0.02
Esophagus D0.05ccm	1.3 Gy	< 0.001	0.8 Gy	= 0.11
Heart D1ccm	0.05 Gy	< 0.001	0.01 Gy	= 0.63
Ribs D0.05ccm	2.0 Gy	< 0.001	2.1 Gy	= 0.11

Thoracic Wall D0.05ccm	0.9 Gy	= 0.02	0.7 Gy	= 0.047
Thoracic Wall D1ccm	2.5 Gy	< 0.001	1.8 Gy	= 0.047
Skin D1ccm	1.1 Gy	= 0.002	0.8 Gy	= 0.30
Bronchus D0.05ccm	3.7 Gy (n = 8)	= 0.02 (n = 8)	0.02 Gy (n = 1)	= 1 (n = 1)
Bronchus_PRV D0.05ccm	2.3 Gy (n = 8)	= 0.055 (n = 8)	0.03 Gy (n = 1)	= 1 (n = 1)
